# Exercise health belief model mediates the relationship between physical activity and peer support among Chinese college students: A cross-sectional survey

**DOI:** 10.3389/fpsyg.2023.1103109

**Published:** 2023-02-06

**Authors:** Jiazhi Sheng, Lamei Gong, Jian Zhou

**Affiliations:** ^1^Laboratory of Sports and Health Promotion, School of Physical Education, Sichuan University of Arts and Science, Dazhou, China; ^2^Graduate School of Management, Management and Science University, Shah Alam, Malaysia; ^3^School of Health Caring Industry, Sichuan University of Arts and Science, Dazhou, China

**Keywords:** perceived benefits, perceived barriers, perceived severity, self-efficacy of exercise, gender differences

## Abstract

This study explored the influence of the exercise health belief model and peer support on university students’ physical activity PA and clarified the related mechanism. Three hundred and thirty-six healthy university students (aged 19.4 ± 1.3 years, 166 male and 170 female) from Sichuan University of Arts and Science in China were evaluated by the peer support scale, the health belief model scale for exercise, and the physical activity scale (short volume). The results showed that the male students’ exercise self-efficacy and PA were markedly higher than female university students. Peer support was positively correlated with perceived benefits, exercise self-efficacy, perceived severity, and cues to action, and was adversely associated with perceived objective and subjective barriers. PA was positively correlated with perceived benefits and exercise self-efficacy, and negatively correlated with perceived objective and subjective barriers. Among the components of the exercise health belief model, only exercise self-efficacy was suitable for constructing a structural equation model (SEM) with peer support and PA. The analysis showed that the predictive effect of exercise self-efficacy on PA was more significant than peer support, and exercise self-efficacy played a critical intermediary role. It is worth noting that, in the grouping model, the effect of male college students’ exercise self-efficacy on PA was greater than that of female students, and the model fit of male peer support was better than that of female students. Although the impact of peer support on PA was less than that of exercise self-efficacy and the direct effect of peer support was less than the indirect effect, the impact of peer support on the PA of female university students was higher than that of male university students. This study revealed the impact of exercise self-efficacy and peer support on university students’ PA and suggested that exercise self-efficacy is the main path to promoting university students’ PA, followed by peer support. Peer support could affect university students’ PA not only through direct effects but also through indirect effects. This study also suggested that female university students’ peer support has a higher impact on PA than male students. Therefore, when formulating physical exercise courses in the future, it is necessary to give more peer support to female university students to compensate for their low exercise self-efficacy.

## Introduction

Insufficient PA is a global health crisis. The World Health Organization (WHO) recently estimated that more than 25% of adults and 80% of adolescents worldwide were physically inactive (World Health Organization [WHO], 2022), increasing the risk of them suffering from various chronic metabolic diseases. The latest guidelines emphasized that people aged 18 to 64 should carry out moderate-intensity aerobic PA for at least 75–150 min or 150–300 min or the same amount of PA to maintain better or promote their health. In recent years, the physical quality of Chinese college students has been declining year by year. In past college students’ physical fitness tests, the excellent rate of physical performance was far lower than the proportion of obesity and overweight. The physical condition of college students from Tsinghua University in China was studied (Wang, 2019), and the results showed that the risk of obesity among college students with insufficient exercise was 1.25 times higher than that of students with regular exercise. There was consensus on the factors affecting the PA of teenagers and college students, such as family support factors (Treiber et al., 1991), social support (Ling et al., 2014), and peer support (Sanz-Martín et al., 2022). Many studies have subdivided the influencing factors into external and internal factors, such as interest, physical exercise confidence and skills, and awareness of the exercise health belief model. However, relying only on internal or external factors, it was difficult to comprehensively and scientifically explain their PA. Relevant studies in this field should be paid adequate attention to explore the relationship between family support/peer support, exercise health belief model and PA.

### Peer support and PA

Peers play an essential role in adolescents’ behavior changes. Peer support mainly includes physical exercise attitude and exercise companionship; through physical exercise, adolescents can make friends and establish a significant correlation between peer relationships and motivation to participate in sports (Klint and Weiss, 1987). Peers can influence teenagers’ exercise experience and motivate them (Agans et al., 2018), uninfluenced by the origin country of the teenagers, whether China (Luo et al., 2011) or the United States (Ling et al., 2014). With increasing age, peer support has an increasingly significant impact on PA. For example, Rajbhandari-Thapa et al. (2022) reported that, with the improvement of a supportive school environment and peer support, students’ PA increased (Rajbhandari-Thapa et al., 2022). Through a survey of families and adolescents from 74 countries, scholars found that Western Pacific countries have the highest correlation between parental support and adolescents’ PA, while in Southeast Asia, peer support has the highest correlation with adolescents’ PA (Khan et al., 2020). Treiber et al. (1991) reported that gender and ethnic factors could affect PA through family and peer support. They found that white women’s physical exercise and PA of leisure time positively correlated with their friends’ support. In white men, PA and total energy consumption correlated with support from family and friends, and the PA of black women positively correlated with family support. The PA of black men positively related to family support and support from friends (Treiber et al., 1991). Based on the above research, we propose hypothesis 1: Peer support is significantly positively correlated with PA, and is affected by gender.

### Exercise health belief model and PA

The health belief model (HBM), as a framework theory, can predict and explain PA (Rahmati-Najarkolaei et al., 2015), and is widely used in the research of people from different demographics, such as PA of older adults (Gristwood, 2011; Kaushal et al., 2021) or women (Hosseini et al., 2017; Shafieian and Kazemi, 2017). The health belief model for exercise evolved from the HBM and can effectively predict or explain PA. For example, Soleymanian et al. (2014) developed and evaluated a tool based on the HBM to estimate the factors affecting exercise behavior and preventing pre-menopausal osteoporosis in women, indicating that the scale has excellent reliability and validity (Soleymanian et al., 2014). Our previous research structured a new scale based on the HBM; we verified the reliability and validity of the exercise health belief model and explored the relationship between the internal components of the model. Further factors can be used to explain PA’s impact on college students (Gong and Sheng, 2022). Exercise self-efficacy and perceived barriers are the two most essential elements in the model. Exercise self-efficacy is a person’s confidence in implementing physical exercise, which is not related to the exercise level or exercise skills, and indicates the possibility of an individual adopting a specific behavior to maintain or promote health (Bandura, 1977). A previous study reported that increased self-efficacy significantly improved the PA of Chinese immigrant women (Cho and Lee, 2013).

In contrast, in a survey of college students from Chongqing (China), Ouyang et al. (2019) found that self-efficacy played a significant mediating role between body image and exercise participation (Ouyang et al., 2019). A cross-sectional survey of 1296 junior middle school students in Shanghai (China) was reported by Yang (2016); he found that parents’ support for teenagers’ exercise behavior do not affect their self-efficacy and satisfaction in participating. It was worth noting that the higher the peer support for exercise behavior, the easier it was for teenagers to overcome barriers and participate in physical exercise. In the meantime, he suggested that self-efficacy mediates the relationship between peer support and PA. Dong (2017) surveyed 1,945 young students aged 13–18, and found that girls had a higher level of social support, while boys had a higher health belief in physical fitness (Dong, 2017). Based on the above research, we propose hypothesis 2: There are differences in health belief model for exercise, peer support, and PA between male and female university students, and hypothesis 3 is proposed as follows: Exercise self-efficacy acts as a mediator on the association between peer support and PA.

This study used the peer support scale, the health belief model scale for exercise, and the PA scale to investigate university students from China, compare and analyze the differences between male and female students in peer support, exercise health belief and PA, and investigate the role of exercise health belief in the relationship between peer support and PA of college students.

## Participants and methods

### Participants

This study used random sampling to select 400 university students from a Western China public university to complete the test voluntarily. The criteria for the inclusion of participants in this study were as follows: (1) full-time college students without any action and physical exercise disorders; (2) no mental illness or mental disorder; and (3) the ability to independently understand language and words. At each instance, the teacher provided the instructions in detail, and then, the participants filled in the questionnaire and submitted it to the teacher on the spot.

### Data collection

The data collection period of this study was from May to June 2022. Two staff members independently reviewed the collected questionnaires. Incomplete or apparent contradictions in the questionnaires were regarded as invalid questionnaires. There were 192 questionnaires from the School of Physical Education (including 25 invalid questionnaires) and 208 questionnaires from the School of Teacher Education and the School of Health Caring Industry (including 39 invalid questionnaires), resulting in 336 final valid questionnaires (effective rate of 84%).

### Ethical statement

The Human Academic Ethics Committee of the Sichuan University of Arts and Science approved this study (approval No. 2022SASULL–002) following the Declaration of Helsinki. All the participants completed the questionnaire after signing the informed consent, and they volunteered to participate in the survey. The data were collected with the intention to hide the participant’s name and declared (before completing the questionnaire) that these data were only available for research purposes.

### General demographic information survey

The participants’ age, sex, BMI (body mass index), and monthly family income were counted and scored. Our previous study detailed the rules (Gong and Sheng, 2022).

### PA survey

The International PA Questionnaire–Short Volume (Chinese Version) (Bassett, 2003) was used to calculate the PA of the participants. The questionnaire required the participants to recall the physical activities (including high, moderate, and low-intensity) carried out in the previous 7 days. The total amount of high-intensity, moderate-intensity, and low-intensity activities was calculated by multiplying the corresponding number of activities carried out per day with the number of days of the type of PA. Previous relevant investigations have confirmed that the scale has good reliability and validity in a survey of the Chinese population (Macfarlane et al., 2007; Deng et al., 2008).

### Peer support survey

The social support scale for physical exercise (Sallis et al., 1987) was used to estimate peer support for PA. The current study drew on a report by Chen et al. (2017) which assessed peer support for physical exercise of middle school students from Fuzhou (China), and ultimately retained five independent factors, such as “changing their schedule to exercise with me.” A Likert 5-point scoring method indicated that attitudes ranged from 1 (very disagree) to 5 (very agree). The internal consistency coefficient α was from 0.7 to 0.80 and the coefficient was from 0.80 to 0.90, both of which are considered good reliability (Terwee et al., 2007). The internal consistency coefficient α of peer support in the current study was 0.850, indicating good reliability. Using exploratory factor analysis, we found that the factor loadings of the five measurable variables were 0.811, 0.837, 0.798, 0.785, and 0.736, indicating that the scale had reliable validity.

### A survey of exercise health belief model

The exercise health belief scale based on the HBM can more effectively predict or explain PA. Wu et al. (2020) developed a scale comprising six dimensions for Chinese adults (Wu et al., 2020), and the internal consistency coefficient of this scale α (0.609) is considered acceptable. The scale consists of perceived benefits (3 measurable variables), perceived subjective barriers (3 measurable variables), perceived objective barriers (4 measurable variables), exercise self-efficacy (5 measurable variables), perceived severity (3 measurable variables), and cues to action (3 measurable variables). The Likert 5-point scoring method indicated that attitudes ranged from 1 (very disagree) to 5 (very agree). At first, the corresponding dimensions were perceived benefits 0.628, perceived objective barriers 0.713, perceived subjective barriers 0.628, exercise self-efficacy 0.801, perceived severity 0.676, and cues to action 0.838. Previous research indicated that only those measured variables whose Cronbach’s alpha coefficients are greater than or equal to 0.6 should be retained in the scale (Bagozzi and Yi, 1988). After removing the factors with a coefficient less than 0.6, the factors constituting the exercise health belief model scale were as follows: perceived benefits (3 measurable variables), perceived subjective barriers (2 measurable variables), perceived objective barriers (3 measurable variables), exercise self-efficacy (4 measurable variables), perceived severity (3 measurable variables), and cues to action (3 measurable variables). The internal consistency coefficient α of the exercise health belief model in the current research was 0.762, perceived benefits 0.712, perceived objective barriers 0.649, perceived subjective barriers 0.637, exercise self-efficacy 0.798, perceived severity 0.665, and cues to action 0.758. This showed that the scale had good reliability. The factor load analysis of the measurable variables included: perceived benefits (3 measurable variables were 0.761, 0.839, and 0.711, respectively), perceived subjective barriers (2 measurable variables were 0.690, and 0.630, respectively), perceived objective barriers (3 measurable variables were 0.711, 0.645, and 0.697, respectively), exercise self-efficacy (4 measurable variables were 0.720, 0.827, 0.662, and 0.654, respectively), perceived severity (3 measurable variables were 0.693, 0.832, and 0.732, respectively), and cues to action (3 measurable variables were 0.735, 0.850, and 0.791, respectively). The above data showed that the scale had good reliability, which was consistent with the conclusion of our previous research report (Gong and Sheng, 2022).

### Data analysis

SPSS 23.0 and Amos 23.0 were used to analyze the data. Mean ± standard deviation was used to summarize the participants’ demographic characteristics, peer support of physical exercise, exercise health belief characteristics and PA. Independent *T*-tests were used to evaluate the differences between male and female students in peer support, exercise health belief and PA. Variance analysis was used to evaluate the interaction between peer support, exercise health belief and PA. Amos 23.0 software was used to evaluate the hypothetical model fit, the direct/indirect relationship among peer support, the constituent elements of the exercise health belief model and PA. We followed the two-step method proposed by Gerbing and Anderson (1988): The first step used confirmatory analysis to evaluate the effectiveness of the measurement model (Gerbing and Anderson, 1988) and the second step used the SEM analysis to measure the fitness and path coefficient of the model. According to the suggestions of scholars, CMID/DF, GFI, AGFI, RMSEA, SRMR, TLI, IFI, and CFI were used to estimate the model’s fitness, and the difference was statistically significant at a *P*-value of < 0.05.

## Results

### Characteristics of participants

The survey results showed that the PA level of male university students was significantly higher than that of female students in high-intensity and moderate-intensity; but there was no significant difference in low-intensity PA (Tables 1, 2).

**TABLE 1:** Basic information of participants (M ± SD).

Items	Over all (336)	Men (160)	Women (176)	*P*-value
Age (year)	19.4 ± 1.3	19.4 ± 1.2	19.5 ± 1.4	0.363
BMI (kg/m^2^)	21.0 ± 2.4	21.8 ± 2.1	20.3 ± 2.4	0.000
Incoming (Yuan/ month)	3.7 ± 2.3	4.1 ± 2.5	3.2 ± 2.0	0.000

**TABLE 2:** Physical activity (PA) of college students.

Items	H_Q1_	H_Q2_	H_Q3_	M_Q1_	M_Q2_	M_Q3_	L_Q1_	L_Q2_	L_Q3_
All (336)	0.0	120.0	247.0	30.0	120.0	240.0	140.0	210.0	420.0
Male (160)	90.0	180.0	300.0	60.0	180.0	360.0	150.0	245.0	420.0
Female (176)	0.0	40.0	180.0	12.5	60.0	195.0	101.3	210.0	420.0
*P*		0.000			0.001			0.310	

Q1, first quartile; Q2, second quartile; Q3, third quartile; H, high-intensity PA; M, moderate-intensity PA; L, low-intensity PA.

**FIGURE 1:**
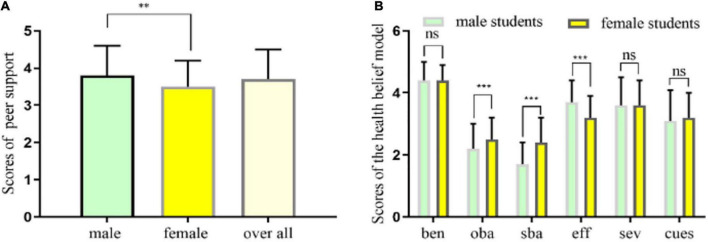
**(A,B)** Male college students’ sup was significant higher than female, the scores of sba and oba was significant lower than female, and eff-e was significant higher than female. ben, perceived benefits; sba, perceived subjective barriers; oba, perceived objective barriers; sev, perceived severity; cues, cues to action; eff-e, exercise self-efficacy. **p < 0.01, ***p < 0.001, ns, there was no statistical difference between two groups.

To compare the differences between male and female university students in peer support and exercise health belief, we used an independent sample *T*-test to evaluate. We found that male university students’ peer support was significantly higher than that of female students. Among the constituent elements of the health belief model scale for exercise, male university students’ scores of perceived subjective and objective barriers were lower than those of female students, and their exercise self-efficacy was significantly higher than that of female students (Figures 1A, B).

### Relationship between peer support, exercise health belief model, and PA of college students

Through Pearson correlation analysis, we found that peer support significantly correlated with perceived benefits, exercise self-efficacy, perceived severity, and cues to action, and was negatively associated with perceived objective and subjective barriers. It significantly correlated with high, moderate, and low-intensity PA. Perceived benefits were significantly associated with moderate-intensity PA, and perceived objective barriers and perceived subjective barriers were significantly associated with high, moderate, and low-intensity PA (and the correlation coefficient between perceived subjective barriers and PA level parameters were more significant than that of perceived objective barriers); exercise self-efficacy was significantly positively correlated with the PA. Perceived severity was significantly correlated with moderate-intensity PA. No significant correlation was found between cues to action and PA (Table 3).

**TABLE 3:** Relationship between peer support, Health Belief Model Scale for Exercise, and physical activity (PA) (*n* = 336).

Items	sup	ben	oba	sba	eff-e	sev	cues
ben	0.186**	−	−	−	−	−	−
oba	-0.432***	-0.114*	−	−	−	−	−
sba	-0.376***	-0.172**	0.473***	−	−	−	−
eff-e	0.391***	0.207***	-0.426***	-0.641***	−	−	−
sev	0.161**	0.129*	-0.079	-0.137*	0.168**	−	−
cues	0.308***	0.069	-0.100	0.050	0.186**	0.165**	−
H	0.272***	0.141**	-0.274***	-0.358***	0.429***	0.049	0.020
M	0.251***	0.142**	-0.285***	-0.336***	0.399***	0.129*	0.061
L	0.119*	0.085	-0.131*	-0.189**	0.243***	0.050	0.024

*p < 0.05, **P < 0.01, ***P < 0.001. sup, peer support; ben, perceived benefits; oba, perceived objective barriers; sba, perceived subjective barriers; eff-e, self-efficacy of exercise; sev, perceived severity; cues, cues to action. H, high-intensity PA; M, moderate-intensity PA; L, low-intensity PA.

**FIGURE 2:**
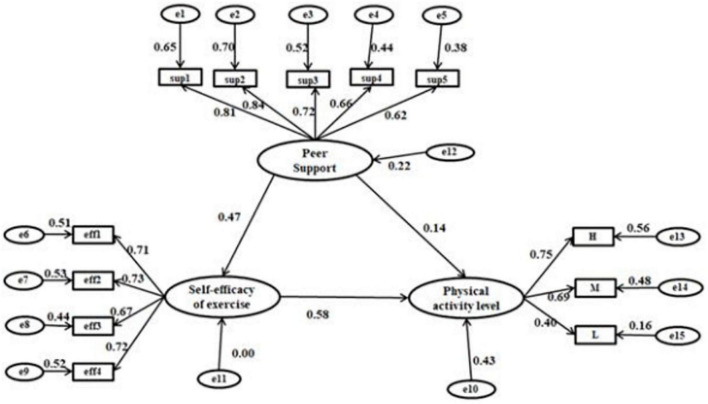
The overall structural equation model. Model–fit indices were statistically acceptable (CMID/DF = 2.778, GFI = 0.926, AGFI = 0.892, RMSEA = 0.073, *p* = 0.001, CFI = 0.926, IFI = 0.906).

**FIGURE 3:**
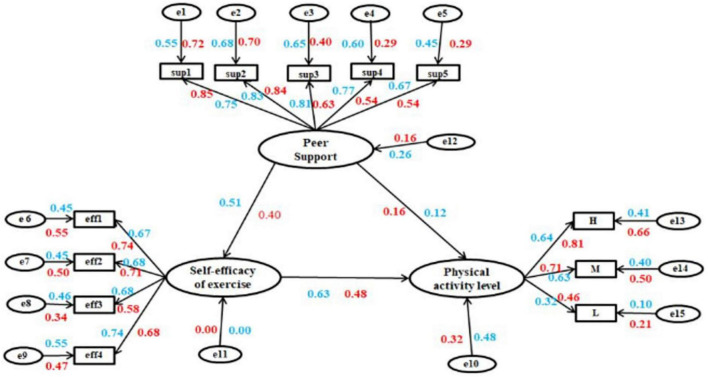
The grouping structural equation model. The light blue represents the data of male college students, and red represents the data of female. In the male group: CMID/DF = 1.948, GFI = 0.905, AGFI = 0.855, RMSEA = 0.073, *p* = 0.001, CFI = 0.929, IFI = 0.932, RMSEA = 0.05–0.08, it indicated model-fit was good. In the female group: Model–fit indices was acceptable. CMID/DF = 2.204, GFI = 0.904, AGFI = 0.853, RMSEA = 0.083, *p* = 0.001, CFI = 0.908, IFI = 0.911, RMSEA = 0.08–0.10, it indicated that model-fit was acceptable.

Structural equation model has been used widely in the research of social sciences. For example, it was used to assess the association between self-efficacy, social support, and the PA level of Peking University students in China (Wang et al., 2019). A survey and SEM of 2206 fourth-grade students and their parents in Turkey found that (Mirzayi et al., 2021) students’ self-efficacy was significantly correlated with moderate and high-intensity PA, and negatively correlated with sedentary behavior. Therefore, we initially attempted to build an SEM of the health belief model for exercise, the peer support, and the PA of university students. After further statistical analysis, we found that only exercise self-efficacy significantly affected university students’ PA. Hence, we constructed an SEM of exercise self-efficacy, peer support, and university students’ PA (see Figure 2), and a grouping model of male and female university students (see Figure 3). According to the total SEM, the effect of peer support on university students’ PA was 0.14, and the indirect effect was 0.27 (0.47 * 0.58). The indirect effect was more significant than the direct effect, suggesting that exercise self-efficacy mediated the relationship between peer support and PA. The effect of exercise self-efficacy on university students’ PA was 0.58, and the effect of exercise self-efficacy on PA was substantially more significant than that of peer support (see Figure 2). Further analysis showed no interaction between peer support and exercise self-efficacy in the relationship between PA. The exciting discovery was that the results of the grouping model showed that the effect of exercise self-efficacy on PA in the male group was 0.63, which was more significant than 0.48 in the female group. The model fitting of the SEM of male students was better that of female students (Figure 3). Although the impact of peer support on PA was less than that of exercise self-efficacy, and the direct effect of peer support was less than the indirect effect, the impact of peer support on the PA of female college students was higher than that of male students.

## Discussion

The main findings of this survey are as follows: (1) Male university students’ peer support was markedly higher than that of female university students, and moderate to high-intensity PA was significantly higher than that of female university students. In terms of the constituent elements of the exercise health belief model, compared with female university students, male university students had higher exercise self-efficacy and lower perceived subjective barriers and perceived objective barriers, and other factors had no significant difference. (2) Peer support was significantly positively correlated with perceived benefits, exercise self-efficacy, perceived severity, and cues to action. It was negatively correlated with perceived subjective barriers and perceived objective barriers, and was significantly positively correlated with high-intensity, moderate-intensity, and low-intensity PA. Perceived subjective and objective barriers were negatively correlated with high-intensity, moderate-intensity, and low-intensity PA. Perceived benefits and exercise self-efficacy were significantly correlated with high-intensity and moderate-intensity PA. (3) Based on the SEM analysis, we found that peer support and exercise self-efficacy significantly affected university students’ PA, and exercise self-efficacy was higher than that of peer support. Through further grouping, we found that female university students’ peer support impact on PA was higher than that of male students, and the impact of exercise self-efficacy on PA was lower than that of male students, reflecting significant gender differences.

### Relationship between demographic information and PA

Insufficient or lack of PA is a complex and long-term problem that needs to be evaluated for many factors. In the process of individual development, boys were more active than girls, and their level of PA was higher than girls. Hopkins et al. (2022) found that the self-concept related to physical exercise was closely related to the physical exercise of female adolescents through a comprehensive analysis. The study emphasized the need for improvement in macro and micro aspects to improve adolescents’ PA and promote fairness (Ricardo et al., 2022). The most significant barriers for girls to participate in PA most likely came from personal factors, such as the values and support of family and friends, a safe environment and the opportunity to participate in PA in school (Guthold et al., 2022). In our study, we found that male university students’ PA was higher than that of females, consistent with the overall gender differences in PA in adolescence. A previous study showed that in exercise practice at the middle school stage, female physical education (PE) teachers were more popular with male students in middle school, and male PE teachers were more popular with female students (Cruz et al., 2021). This investigation affirmed that we should pay attention to the course content, and create a good relationship between teachers and students in the PE curriculum.

### The relationship between peer support and the PA of college students

With increasing age, the effect of peers on PA gradually became more remarkable than that of family and had significant gender characteristics. Hong et al. (2020) surveyed 61,429 school-age children (aged 6–18, boys accounted for 50.7%) and their families in Shanghai (China). They found that boys were more active than girls, and the survey also showed that girls were more vulnerable to family factors than boys. The research suggested that paying attention to the role model of parents’ physical exercise may be more important than other factors (Hong et al., 2020). Khan and Uddin (2020) investigated Malaysian adolescents (aged 11–17, boys accounted for 49%) and their families, where 15.3% reported having a positive lifestyle, of which 22% were boys and 8.8% were girls, 47% reported having a higher level of peer support, and 31% reported having higher parental support (Khan and Uddin, 2020). Higher parental support significantly correlated with the positive lifestyle of boys, and girls with higher parental support had a higher probability of a positive lifestyle, while girls aged 11–14 years had a more significant correlation between peer support and a positive lifestyle. Peterson et al. (2013) conducted a cross-sectional survey of sixth-grade students from South Carolina and found that the instrumental social support of parents significantly correlated with girls’ PA. It was worth noting that the emotional support of parents negatively correlated with the PA of girls, and self-efficacy mediated the relationship between parents’ instrumental social support and boys’ PA (Peterson et al., 2013).

Kulik et al. (2014) found that increasing peer support for 4 months significantly increased the PA of American girls, who eventually lost weight. Gruber (2008) surveyed American college students (North Carolina State University) and found that compared with men, women’s reports showed that their healthy eating behavior and exercise habits were more supported. The study further found gender differences in the composition of peers. For example, when more than half of their friends were men, female students received more support in physical exercise and good eating habits. When more than half of their peers or friends were female students, male students reported the highest level of peer support. On the other hand, when more than half of their peers were men, male students’ exercise habits received a higher evaluation. When most of their peers or friends were female students, female students reported the highest level of peer support. In our study, male university students’ peer support was significantly higher than female students. However, the peer support of female university students was higher than that of male university students in the impact of PA, and self-efficacy played a mediating role in the relationship between peer support and PA.

### Effect of exercise self-efficacy on the relationship between peer support and the PA of college students

Exercise self-efficacy is the confidence to perform physical exercise behavior, which was compared to internal motivation. However, peer support could be compared to the external support environment for individual physical exercise and termed external cause. Self-efficacy and perceived barriers were the two action items that affect exercise behavior in the exercise health belief and were the main factors that predict or explain the level of PA (Fletcher and Banasik, 2001). Better exercise compliance was significantly related to increased exercise self-efficacy. A cross-sectional investigation of 101 menopausal women from Queensland was conducted by Barnett and Spinks (2007). This survey showed that participants with high self-efficacy in the preceding 7 days had a significantly greater capacity to overcome barriers to physical exercise than those with low self-efficacy. The study suggested that improving exercise self-efficacy helps to reduce perceived barriers to exercise and helps to assist menopausal women in obtaining health benefits from physical exercise. As an essential predictor of exercise behavior adoption and maintenance, exercise self-efficacy could express a person’s confidence in successfully performing physical exercise (Fletcher and Banasik, 2001), but it could also significantly predict the PA level of sedentary individuals (McAuley and Jacobson, 1991). For example, Hamilton et al. (2017) found that self-efficacy became the main predictor (intrinsic motivation) of violent adolescent sports, followed by support (extrinsic support) of friends; furthermore, friends’ support and self-efficacy were interrelated (Hamilton et al., 2017). In the youth group with high self-efficacy, the intention of PA had nothing to do with peer support. In the group with low self-efficacy, the physical exercise activities of peers made up for the lack of self-efficacy. The research suggested that, for the group with poor self-efficacy, giving enough peer exercise peer support is an effective way to promote their participation in physical exercise. Spence et al. (2010) surveyed young people from Alberta (Canada) and found that self-efficacy had a stronger correlation with girls’ PA (Spence et al., 2010). In our research, exercise self-efficacy was the most significant factor in explaining PA, whether in the overall model or the group model, which is consistent with the conclusion of Hamilton et al. (2017). Furthermore, in our study, a stronger correlation was found between exercise self-efficacy and PA of male college students (0.63 vs. 0.48) in the group analysis, which is significantly different from the findings of Spence et al. (2010). Our study also showed that, in the relationship between peer support and PA, the correlation coefficient of female university students was higher than that of male university students.

Seo and Ha (2019) conducted a cross-sectional study of college students from South Korea. They found that the factors affecting the PA of male college students were exercise self-efficacy and personal economic level. In contrast, the factors affecting the PA of female college students were exercise self-efficacy, subjective health status, activity-related emotions, and peer support (Seo and Ha, 2019). It suggested that gender differences should be considered when focusing on exercise self-efficacy and plans. Xue-Liu and Mu (2021) surveyed 801 college students at a university in Chongqing in Southwest China; using the college students’ health belief model scale for exercise and PA rating scale (PARS-3) (Xue-Liu and Mu, 2021), multiple regression analysis showed that perceived exercise benefits, self-efficacy, and exercise volume were positively correlated. The effect of physical health belief-based self-efficacy on exercise volume was greater than that of perceived exercise benefits. Aware of the severity of illness and weakness, their research suggested that the self-efficacy of physical evaluation can strongly predict the amount of exercise of college students. Their data processing used a multiple regression equation related to the single equation model method. Regression analysis can only deal with dominant variables, while SEM can more effectively find potential variables. A multiple regression model has only one dependent variable and is unidirectional, while SEM could be unidirectional or bidirectional. A multiple regression model, was the basis of their study. However, in terms of statistical methods, SEM has its advantages. In our study, using SEM, we found that only exercise self-efficacy significantly correlated with college students’ PA in exercise health belief. We also found that exercise self-efficacy significantly mediated between university students’ peer support and PA.

### Limitations and future directions

Although this study investigated peer support, exercise health beliefs, and PA of Chinese university students, analyzed exercise self-efficacy’s significant mediating role in the relationship between peer support and PA, and initially explored the practical path to promote university students’ extracurricular physical exercise, it still had the following limitations. First, the sampling method used in this study was random sampling, which may not accurately represent the situation of the whole school. Therefore, the stratified sampling method should be used in future research. Second, this investigation was a cross-sectional survey, and the data obtained could not provide conclusive evidence of causality between variables. Therefore, future studies should include longitudinal cohort studies and further evaluate the impact of increasing health education on exercise health belief and PA. Third, the data collected in this study were only from one public university in Western China, which means that the conclusions obtained in this study have significant regional characteristics, and need to be carefully inferred when applying them to colleges and universities in other regions of China. Therefore, future research should fully consider more geographical factors (coastal cities in eastern China, cities in the southeast, cities in the north, etc.), the attributes of colleges (public vs. private), and larger sample sizes.

## Conclusion

This study revealed the impact of exercise self-efficacy and peer support on university students’ PA, suggesting that exercise self-efficacy is the main path to promoting university students’ PA, followed by peer support. Peer support affects university students’ PA not only through direct effects but also through indirect effects. This study suggests that female university students’ peer support impacts PA more than males. This research finding provides ideas for more effective future development and guidance of physical exercise practice. When formulating physical exercise courses in the future, it is necessary to plan for more peer support for female college students to compensate for their inadequate exercise self-efficacy.

## Data availability statement

The raw data supporting the conclusions of this article will be made available by the authors, without undue reservation.

## Ethics statement

The studies involving human participants were reviewed and approved by Sichuan University of Arts and Science (approval No. 2022SASULL–002). The patients/participants provided their written informed consent to participate in this study.

## Author contributions

JS and LG: conceptualization, data curation, writing—original draft preparation, and resources. JS: methodology and writing—review and editing. LG and JZ: investigation. All authors have read and agreed to the published version of the manuscript.
